# Schottky Barriers Based on Nanoporous InP with Gold Nanoparticles

**DOI:** 10.1186/s11671-016-1399-4

**Published:** 2016-04-14

**Authors:** Tetyana Barlas, Mykola Dmitruk, Nataliya Kotova, Sergii Mamykin

**Affiliations:** V. Lashkaryov Institute of Semiconductor Physics, National Academy of Sciences of Ukraine, 41, Prospect Nauky, Kyiv, 03028 Ukraine

**Keywords:** Porous indium phosphide, Photocurrent, Current-voltage characteristics, Surface plasmons, Gold nanoparticles, 73.20.Mf, 78.67.Bf, 73.30+y

## Abstract

Schottky barrier structures based on nanoporous InP with inclusion of Au nanoparticles and evaporated semitransparent Au film have been made. The spectra of short-circuit photocurrent in the visible range and current-voltage characteristics have been measured. Prepared structures are characterized by increased photocurrent due to the microrelief interface and surface plasmon excitation in gold nanoparticles as well as increased surface recombination especially in the short wavelength region.

## Background

Nanocomposites, which are porous semiconductors with metal inclusions, and structures based on them seem to be promising for use in optoelectronics, photovoltaics, sensorics, etc. due to their optical and electronic properties which are different from the bulk materials, the possibility of simple tuning of their properties, and the large surface-to-volume ratio. Over the last decades, different methods to fabricate porous III-V semiconductors have been developed, and mainly, electrochemical etching was used [1–3]. It should be noted that porous InP attracts the interest of many researchers as a semiconductor with direct band gap and also as a material with quite perfect pore structure and possibility of easy change of pore parameters for a specific application. Therefore, many modifications of electrochemical etching are proposed, for example, the two-step anodic-cathodic reaction method [4]. Fabrication of the inclusions of noble metals such as Pt and Au inside the pores in InP and GaP in different forms (nanodots, nanoparticles, nanotubes) is reported in [5–10], and to obtain uniform deposition of metal, pulsed electroplating was used [5–9]. Local plasmon excitation in metal nanoparticles leads to localization, concentration, and local enhancement of electromagnetic fields in their vicinity, thus causing an enhancement of many photophysical phenomena such as photoluminescence, infrared absorption, Raman scattering [10], and photocurrent of barrier structures (plasmonic photovoltaics) [11]. A photoelectric conversion device based on an InP porous structure was proposed in a few papers [9, 12], utilizing the large surface area inside pores and the low reflectance from the porous surface. The initial enhancement of the photocurrent response which is mostly due to decreased light reflection and the redshift of the absorption edge of the photocurrent spectra are observed [13]. Photocurrent spectroscopy and photoluminescence measurements show that the porous film behaves like an absorbent layer [13], and surface states and the prolonged depletion region decrease photocurrent. To decrease the effect of the prolonged depletion region and to form Schottky barrier, Pt nanoparticles have been used [9], and improvement in photocurrent has been shown. To further increase photocurrent, we propose to use Au plasmon-active nanoparticles which can increase barrier height like the mentioned Pt nanoparticles and also can increase light absorption due to surface plasmon excitation. In this paper, a short report on the possibility to use Au nanoparticles embedded into porous InP for the photocurrent enhancement of Au/InP Schottky barrier is presented.

## Methods

Porous InP was fabricated by electrochemical etching of n-type (111) InP wafers with dopant concentration 10^16^ cm^-3^ in aqueous 5% HCl solution in the galvanostatic regime. The current density and etching time were varied in the range of 2-100 mA/cm^2^ and 1-30 min respectively. Au nanoparticles were embedded in the pores from the water solution of AuCl_3_ salt by electrolysis.

The fabricated porous layers and nanocomposites with metal inclusions were structurally analyzed by scanning electron microscopy (SEM) using a JEOL 6700 instrument (with a resolution of 1 to 2.2 nm). The elemental analysis of the fabricated composites was carried out with a microanalyzer for energy dispersive X-ray spectroscopy (EDAX) attached to the microscope used. We investigated both the surfaces and, even more informatively, the fresh cleavages of the porous layers.

Photosensitive surface barrier structures Au-layer/composite have been made by thermal evaporation in vacuum of thin semitransparent Au film. Photoelectric and electric properties of structures have been studied with help of short-circuit photocurrent spectra in the 0.4-1.0 µm spectral range and forward/backward current-voltage characteristics.

## Results and Discussion

**Fig. 1:**
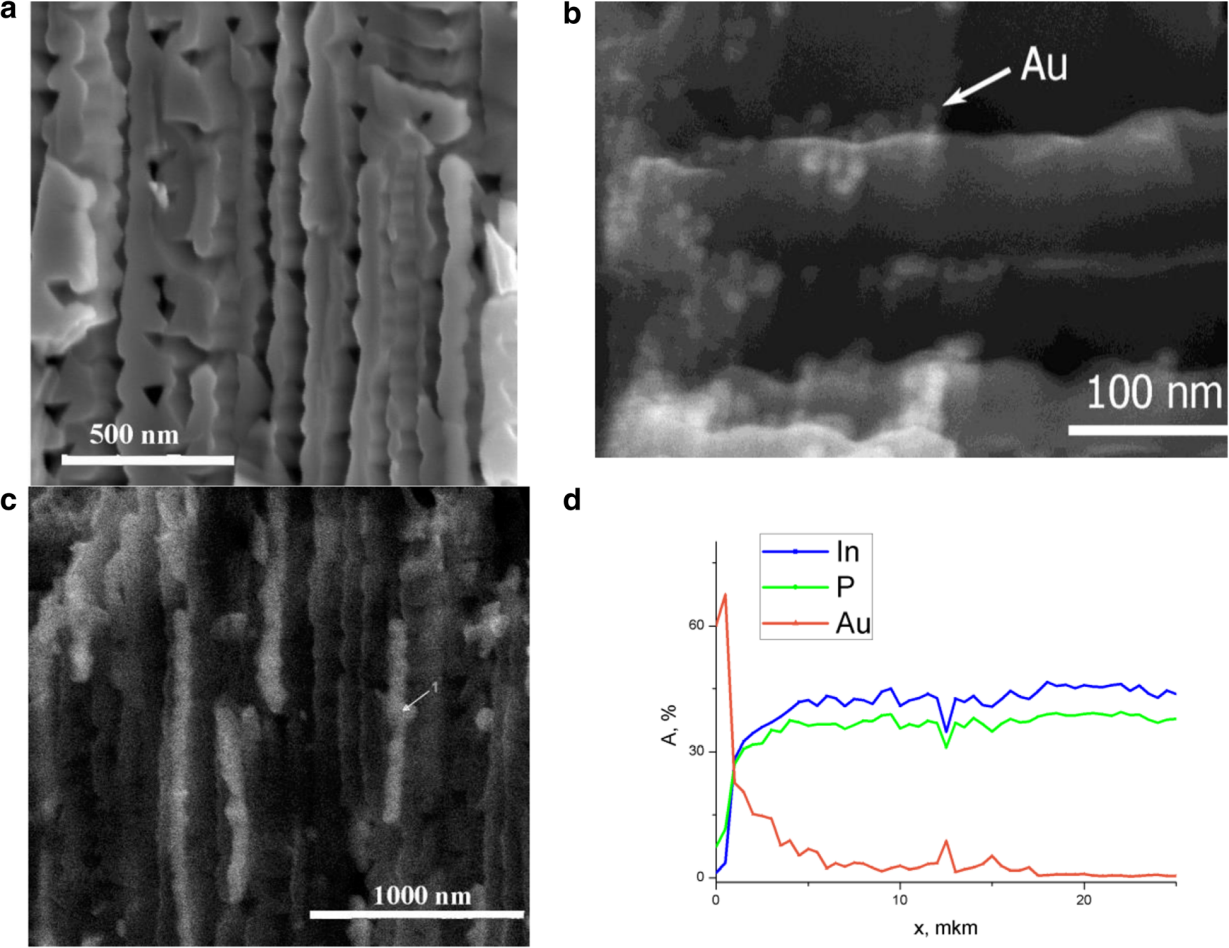
SEM images of cross sections of porous InP without Au nanoparticles (**a**) and with different amounts of embedded Au nanoparticles (**b**, **c**), and element composition of the porous layer as a function of the distance from the surface (**d**) for the porous layer with high concentration of Au nanoparticles

Porous semiconductors have nanosized pores/crystallites which could be used as a template for a composite with metal nanoparticles for optoelectronic and photovoltaic applications. Based on our previous research [2, 10], we fabricated porous InP (Fig. 1) by electrochemical etching of n-type (111) InP wafers with a dopant concentration of 10^16^ cm^−3^ in aqueous 5 % HCl solution in the galvanostatic regime. The current density and etching time were varied in the range of 2–100 mA/cm^2^ and 1–30 min, respectively. The obtained porosity was in the range of 10–70 % and pore size 30–200 nm. Au nanoparticles were embedded in the pores from the water solution of AuCl_3_ salt (Au^3+^ ion concentration was 0.01–1 g/L) by electrolysis.

As confirmed by the results of the SEM studies, porous layers are partially ordered systems of cylindrical cavities or embedded tetrahedrons demonstrating a horizontal-plane correlation between the neighboring pores. Pores are oriented perpendicular to the sample surface. The porous layer shown in Fig. 1 has been fabricated in 5 % HCl aqueous solution at the temperature *T* = 25 °C. The current density was 20 mA/cm^2^, and the etching time was 15 min. In such etching process, pores with a diameter of 100 nm have been created. The porosity of the layer can be estimated as 40 %. The thickness of the porous layer was 20 μm.

Electrodeposition of Au was performed at room temperature *T* = 25 °C, and voltage *U* = −2 V was applied; the duration of the process was 30 min. Au^3+^ ion concentration was 0.1 g/L for the sample with a small amount of Au in the porous layer (Fig. 1b) and 0.4 g/L for the sample with a big amount of it (Fig. 1c). The surface of the samples is partially decorated with metal particles, whereas the cleavage image exhibits that the metal nanoparticles also fill the pores (Fig. 1b–d). As evidenced by EDAX analysis of the nanocomposite, gold is indeed present on the surface as well as inside the pores. Accurately determining the amount of metal in the pores is difficult because gold is deposited on the sample surface too, but the EDAX measurements (Fig. 1d) show that gold is deposited over the entire thickness of the porous layer (up to 20 μm) and its distribution is non-uniform; the larger amount of gold is concentrated near the surface. In the case of the small amount of gold in the composite, the cleavage SEM image (see Fig. 1b) exhibits that the individual spherical Au particles with a diameter of 10–20 nm are in the inner surface of the pores. With the increase of the deposited gold amount, the pores in the semiconductor are partially filled with metal particles (see Fig. 1c). As a result, the gold nanowires are formed in the pores; such inclusion is marked by an arrow in Fig. 1c. Since inside the pore wires, no tubes are created under such conditions of the deposition process, the question of the percolation of gold inclusions along the pore remains open. The optimum amount of metal in the pores and its location require further investigations. Furthermore, this technique allows to deposit the nanoparticles of other metals, such as platinum for example, which may lead to an increase in the photocurrent of the structure [7–9].

**Fig. 2:**
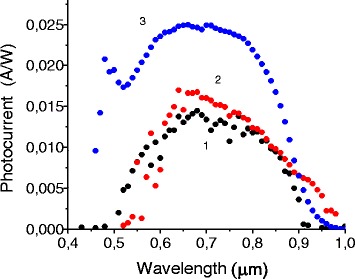
Spectral dependencies of photocurrent of samples based on porous InP without inclusion of Au nanoparticles into the pores (*1*) and with inclusion of their low (*2*) and high (*3*) concentration. The thickness of the barrier contact is 30 nm

**Fig. 3:**
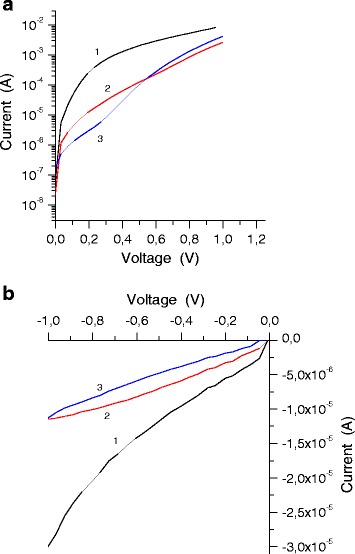
Forward (**a**) and backward (**b**) current-voltage characteristics of the structures based on porous interfaces with different concentrations of Au nanoparticles: (*1*) without nanoparticles, (*2*) with low concentration, and (*3*) with high concentration

Photosensitive surface barrier structures Au-layer/composite have been made, where the composite is a porous InP with Au nanoparticles embedded in the pores. Au barrier contacts with 30-nm thickness and an electrode area of 1.33 mm^2^ have been deposited by thermal evaporation in vacuum. The influence of Au nanoparticles on photoelectric and electric properties of structures has been studied with the help of short-circuit photocurrent spectra (Fig. 2) in the 0.4–1.0-μm spectral range and forward/backward *I*(*V*) (Fig. 3a, b) characteristics.

Porous heterostructures without Au nanoparticles demonstrate quite low photocurrent (Fig. 2 curve 1) in the order of 0.01 A/W. This is connected with the non-optimal porous layer thickness, increased surface state density, and as consequence increased surface recombination. The spectrum demonstrates a short wavelength decreasing in the range of 0.4–0.7 μm which is due to the increased number of photons absorbed near the upper part of the porous layer generating electron-hole pairs far from the InP substrate, thus increasing their probability of recombination during charge separation.

It is seen that the inclusion of Au nanoparticles increases photosensitivity (Fig. 2 curves 2 and 3) of heterostructures compared to structures without nanoparticles due to local (surface) plasmon excitations and, as consequence, increased light absorption [14]. It increases recombination as well, especially in the short wavelength part of the spectrum because the metal/semiconductor interface has usually higher interface state density [15] than the free or passivated semiconductor surface. On the other hand, the barrier height is larger for the Au/InP contact and the charge separation therefore is better. Thus, the total effect is positive, and the photocurrent was increased for structures with incorporated Au nanoparticles.

Inclusion of Au nanoparticles into the pores of the semiconductor positively affects the current-voltage characteristics of heterostructures (Fig. 3a, b). The factor of non-ideality *n* = 3–6 and saturation current *I*_o_ = 0.1–10 A/m^2^ were reduced compared to the porous structures without nanoparticles (*n* = 2–11, *I*_o_ = 2–280 A/m^2^). This can be explained by the increase in the contact area between Au and the semiconductor, mainly due to a contact inside the pores, thus increasing the average barrier height [9] and reducing surface leakage currents.

In general, the large value of *n* and *I*_o_ arise due to the large thickness of the intermediate oxide layer formed by anodic etching, increased density of surface states, and increased area of the electrical contact. The factor of non-ideality *n* > 2 and the presence of the microrelief porous layer, which leads to the concentration of electric fields on the edges of the metal contact, indicate that the main mechanism of current flow is tunneling in combination with space charge-limited conductance [16]. This is evidenced by high values of reverse currents represented in Fig. 3b.

## Conclusions

We used a facile and cost-effective method for the fabrication of a new class of nanocomposite materials, viz., ordered porous III-V semiconductor layers with metal nanoparticles incorporated into the pores. It is shown that this technology followed by Au barrier contact deposition enabled the successful fabrication of Schottky barrier structures. Without Au nanoparticles, the current-voltage characteristics demonstrate the large values of the non-ideality factor and saturation current due to the thick intermediate oxide layer, increased density of surface states, and increased area of interface. Deposition of Au nanoparticles into the pores leads to the photocurrent enhancement because of increased light absorption due to the microrelief interface and surface plasmon excitation in gold nanoparticles. Additionally, the Au nanoparticles in pores lead to the decrease of the saturation currents and non-ideality factor because of improvement of the barrier characteristics. In spite of the increased photocurrent, the surface recombination is also increased especially in the short wavelength region.

## Abbreviations

EDAX: energy-dispersive X-ray spectroscopy
SEM: scanning electron microscopy
